# Relative Mortality Amongst White and Colored Races

**Published:** 1882-01

**Authors:** 


### RELATIVE MORTALITY AMONGST WHITE AND COLORED RACES.

In his annual report for the past year, the Mayor of Savannah draws attention to the great disparity in the percentage of mortality amongst the white and colored races. The annual rate for 1,000 whites for the year 1880 was 19.85, and for 1,000 colored, 45.47 ; these rates being calculated on the United States census tables during the same year. In the Mayor's opinion, the disparity is due to a want of observance of the laws of public hygiene, and to neglect, on the part of many of the lower classes of colored persons, in ministering to the necessities of the sick.—*Gaillard's Medical Journal.*

In a selection from the *Dental Office* and Laboratory, in our Dec. No. the author's name should be *Chupein* not Chapin.

